# The effect of subarachnoid erythrocyte lysate on brain injury: a preliminary study

**DOI:** 10.1042/BSR20160100

**Published:** 2016-07-15

**Authors:** Zi-Huan Zhang, Yan-Ling Han, Chun-Xi Wang, Chen-Hui Zhou, Ling-Yun Wu, Hua-Sheng Zhang, Qiang Chen, Jie-Mei Fan, Meng-Liang Zhou, Chun-Hua Hang

**Affiliations:** * Department of Neurosurgery, Jinling Hospital, School of Medicine, Second Military Medical University, Nanjing 210002, China; † Department of Neurosurgery, Jinling Hospital, School of Medicine, Nanjing University, Nanjing 210002, China; ‡ Department of Neurosurgery, Jinling Hospital, School of Medicine, South Medical University, Nanjing 210002, China

**Keywords:** erythrocyte lysate, inflammation, peroxiredoxin 2, subarachnoid haemorrhage

## Abstract

Abundant erythrocytes remain and lyse partially in the subarachnoid space after severe subarachnoid haemorrhage (SAH). But the effect of subarachnoid erythrocyte lysate on brain injury is still not completely clear. In this study, autologous erythrocytes (the non-lysate group) and their lysate (the lysate group) were injected separately into the cistern magna of rabbits to induce a model of experimental SAH, although the control group received isotonic sodium chloride solution instead of erythrocyte solution. Results showed that vasospasm of the basilar artery was observed at 72 h after experimental SAH, but there was no significant difference between the non-lysate group and the lysate group. Brain injury was more severe in the lysate group than in the non-lysate group. Meanwhile, the levels of peroxiredoxin 2 (Prx2), IL-6 and TNF-α in brain cortex and in CSF were significantly higher in the lysate group than those in the non-lysate group. These results demonstrated that brain injury was more likely to be caused by erythrocyte lysate than by intact erythrocytes in subarachnoid space, and inflammation response positively correlated with Prx2 expression might be involved in mechanism of brain injury after SAH.

## Abstract (precis)

We found that more severe brain injury was caused by subarachnoid erythrocyte lysate, and inflammation associated with Prx2 might be involved in mechanism of brain injury.

## INTRODUCTION

There were several common methods to induce an animal model of experimental subarachnoid haemorrhage (SAH), such as autologous blood injection into cisterna magna [1] or prechiasmatic cistern [2], endovascular perforation [3]. The mechanisms of brain damage after SAH were very complex and not yet fully understood. Early studies mainly focused on cerebral vasospasm (CVS), and recent studies were more inclined to early brain injury (EBI). No matter what kind of study, the damage factor chosen was usually the whole blood or its lysate.

There was a difficult problem during treatment to those patients with severe aneurysmal SAH. Even though aneurysms had been treated, abundant erythrocytes and its lysate in the form of blood clots or free in the cerebrospinal fluid (CSF) still remained in the subarachnoid space, and was hard to be cleared in a short time. Therefore, it was extremely important to understand the role of subarachnoid erythrocytes and their lysate on CVS or on brain injury. Previous studies showed that brain injury was observed when the whole blood lysate was injected into the brain cortex of mice [4] or cisterna magna of rabbits [5], although vasospasm of the basilar artery was also existed when the whole blood lysate was injected into cisterna magna of canine [6]. However, the roles of subarachnoid erythrocytes and their lysate on brain injury are still not absolutely understood.

The peroxiredoxins (Prxs) protein, which has six subtypes (Prx1–6), is expressed widely in animal tissues and serves an antioxidant function associated with removal of cellular peroxides. Among them, Prx2 is the third most abundant protein in the erythrocyte (5.6 mg/ml) [7–9], also selectively expressed in the neuron [10], and mainly distributed in the cytoplasm and the cell membrane. Prx2 would be free into CSF after lysis of erythrocytes. Besides, it has long been known that CVS after aneurysmal SAH causes delayed cerebral ischemia (DCI) and the rate of DCI still remains unacceptably high [11]. And once DCI appears, Prxs would be released extracellularly from neural cells [12], initiated a destructive inflammatory response. What's more, due to neurons more susceptible to ischemia, intracellular Prx2 would be more likely to be released and be finally free in CSF through impaired brain-CSF barrier. Consequently, the source of Prx2 in the subarachnoid space after SAH might be as follows: lysis of subarachnoid erythrocytes and release from damaged neurons. However, this speculation has not been confirmed and the role of extracellular Prx2 after SAH has not yet known.

In this study, a rabbit model inducing SAH was built by injecting autologous erythrocytes or their lysate into cistern magna, to observe CVS and brain injury and explore potential mechanisms.

## MATERIALS AND METHODS

### Animals

Thirty-six adult male New Zealand white rabbits were purchased from the Animal Center of Jinling Hospital, and randomly divided into three groups of 12 rabbits each with a injection into the cisterna magna: the control group (1.5 ml of isotonic sodium chloride solution), the non-lysate group (1.5 ml of intact erythrocyte solution) and the lysate group (1.5 ml of erythrocyte lysate solution). The rabbits were raised in a 12 h dark-light cycle with free access to food and water during the experiment. The feeding room and lab room were kept at 25°C. Three days before SAH, the rabbits underwent the following training (out / into the cage, weighing, fixed in the groove), twice a day, in order to eliminate fear and tension. All experimental protocols used for animals (including all surgical procedures) were approved by the Animal Care and Use Committee of Jinling Hospital and conformed to the Laboratory Animal Care and Use Guidelines of Second Military Medical University.

### Erythrocyte lysate preparation

Three millilitres of arterial blood was withdrawn from the central artery of the ear with a sterile syringe containing heparin (125 u/ml) as anticoagulant. Follow the steps of the past [6], blood was centrifuged for 10 min at 500 ***g*** and the upper plasma/buffy coat was aspirated. Erythrocytes were then similarly washed twice in warm sterile isotonic sodium chloride solution and resuspended in the same solution to 1.5 ml. The intact erythrocyte solution was finally lysed by ultrasonic waves (Ultrasonic Cell Crusher, XO-400S, Xianou Tech) as previously described [13]. The erythrocyte lysate solution was kept at 39°C until use. All above operations were carried out strictly according to the principle of aseptic.

### Experimental SAH

Each rabbit was anesthetized by intravenous injection of diazepam (1.6 mg/kg) and pentobarbital sodium (30 mg/kg), and positioned left-laterally with the head slight extension. The atlantooccipital hair was shaved and the skin was sterilized with 75% ethanol. A butterfly needle (black, 0.7X25TWSB) was inserted percutaneously into the cisterna magna. After withdrawal of 1.5 ml of CSF, the equal amount of intact erythrocyte solution or its lysate solution was slowly injected into the cisterna magna during 2 min. The control group received isotonic sodium chloride solution instead of erythrocyte solution. The rabbits were placed in a head down position for 30 min and then returned to the cage in the lateral position to prevent asphyxia.

The detection time of the experiment was chosen in 72 h based on the following reasons. Firstly, some study showed that under the conditions (incubation at 37°C in an artificial CSF), the rate of haemolysis of erythrocytes was quite slow initially and became more rapid after 4 days’ incubation [14]. Based on this, we supposed that the difference of brain injury in rabbits between the non-lysate group and the lysate group was maximized at 72 h after injection. Secondly, numerous studies showed that EBI was the main factor affecting the prognosis of patients with SAH [15,16], although EBI was the brain damage occurring within the first 72 h after SAH [17,18]. To sum up, we chose 72 h as the detection time of the experiment.

### Perfusion-fixation

At 72 h post-injection, the rabbits were anaesthetized by intravenous injection of diazepam (1.6 mg/kg) and pentobarbital sodium (60 mg/kg). 0.5 ml of CSF was withdrawn from the cistern magna and centrifuged for 10 min at 500 ***g***. The supernatant was kept at -80°C for measuring the levels of inflammatory factors. Perfusion-fixation was then performed as previously described [5]. After the thorax was opened, a cannula was placed in the left ventricle, the descending thoracic aorta was clamped, and the auricula dextra was opened. Perfusion was begun with 500 ml of isotonic sodium chloride solution (4°C), followed by 500 ml of 4% formaldehyde under a perfusion pressure of 120 cmH_2_O in half of the rabbits in each groups. The brain was then removed from the cranium and immersed in the same fixative. The same part of the temporal cortex was obtained from the other half of the rabbits without formaldehyde fixation and immediately froze at -80°C until use.

### Total protein extraction

To extract cortex total protein, the frozen temporal tissue was mechanically lysed in mM Tris pH 7.6, which contained 0.2% SDS, 1% Triton X-100, 1% deoxycholate, 1 mM phenylmethylsulfonyl fluoride (PMSF), and 0.11 IU/ml aprotinin (Sigma). Lysate was centrifuged at 12000 ***g*** for 15 min at 4°C. The supernatant was collected and stored at -80°C until use.

### Evaluation of CVS

The degree of CVS was evaluated by measuring the cross-sectional area of the basilar artery lumen. The formaldehyde-fixed and paraffin-embedded basilar artery sections (4 μm thick) were deparaffinized, hydrated, washed and stained with H&E. Micrographs of the basilar arteries were analysed by using ZEN 2012 SP2 blue edition (ZEISS). Cross-sectional areas of basilar arteries were calculated from the perimeter of the luminal border. For each vessel, three sequential sections (midpoint of proximal, middle and distal) were taken, measured and averaged (Figure 2E). The mean±S.E.M. value obtained for each artery was used as the final value for a particular vessel.

### Evaluation of brain damage

Temporal lobes excised after perfusion-fixation were embedded in paraffin (Figure 2E), and 4-μm-thick coronal sections were examined to detect neural damage in cortical tissue by Nissl staining and immunohistochemistry (IHC).

#### Nissl staining

Coronal sections of temporal lobe were stained with cresyl violet as previously described [19]. Normal neurons had large cell bodies, rich Nissl's bodies in cytoplasm, with one or two big round nuclei. In contrast, damaged cells show shrunken cell bodies, condensed nuclei, dark cytoplasm.

#### IHC

Cleaved caspase-3 expression was evaluated by IHC as previously described [20]. For cleaved caspase-3 staining, coronal sections of temporal lobe were deparaffinized and rehydrated in graded concentrations of ethanol to distilled water. Sections were placed in EDTA buffer (pH 9.0) and heated in a microwave oven until boiling, then cooled at room temperature for 8 min, heated again in a microwave oven at 70°C for 7 min and rinsed in PBS (pH 7.4). Endogenous peroxidase activity was blocked with 3% hydrogen peroxide (H_2_O_2_) for 25 min, followed by a brief rinse in distilled water and a 15-min wash in PBS (pH 7.4). Nonspecific protein binding was blocked by a 30-min incubation in 3% BSA. Sections were incubated overnight at 4°C with a polyclonal anti-cleaved caspase-3 antibody (ab2302, Abcam, UK; 1:150 dilution). Sections were then incubated with goat anti-rabbit biotinylated secondary antibody (Santa Cruz Biotechnology) at room temperature for 50 min. Slides were visualized by incubation with 3,3′-diaminobenzidine (DAB). Nucleus were stained again with Harris haematoxylin for 3 min and blued with ammonia after a temporary treatment with 1% hydrochloric acid alcohol solution (hydrochloric acid:70% alcohol=1:99).

#### Cell counting

One slice from every six serial cuttings in each segment was chosen, and altogether six slices were collected and observed under the light microscope. All surviving neurons (Nissl staining) or positive cells (cleaved caspase-3 staining) in temporal cortex in each section were counted in six microscope fields (magnification ×400) throughout the identical regions of temporal lobe, and the average number per visual field was calculated. All the processes were conducted by two independent, experienced pathologists blinded to the grouping.

### Western blotting analysis

Western blotting analysis was used to measure Prx2 expression in the temporal cortex at 72 h after experimental SAH. Briefly, equal amounts of samples were separated by SDS/12% PAGE and transferred on to polyvinylidene-difluoride membranes (Bio-Rad Laboratories). Then, the membranes were blocked with 5% skimmed milk for 4 h at room temperature, incubated overnight at 4°C with anti-Prx 2 antibody (ab16765, Abcam; 1:1000 dilution), anti-IL-6 antibody (ab154367, Abcam; 1:500 dilution), anti-TNF-α (ab9739, Abcam; 1:3000 dilution) and anti-β actin antibody (ab6276, Abcam; 1:5000 dilution), washed and incubated with second antibody (ab131368, Abcam; 1:5000 dilution), and finally visualized by chemiluminescence.

### Biochemical analysis

The levels of Prx2, IL-6 and TNF-α in CSF were measured respectively using ELISA with the Prx2 kit (CK-E94047R, Calvin Biological Technology), IL-6 kit CK-E80171R, Calvin Biological Technology) and TNF-α kit (CK-E80109R, Calvin Biological Technology) following the manufacturer's instructions.

### Statistical analyses

All data were presented as means±S.E.M. IBM SPSS 19.0 was used for statistical analysis. Statistical comparisons between groups were subjected to one-way ANOVA combined with the Tukey multiple comparison test. Statistical significance was inferred at *P*<0.05.

## RESULTS

### General observations

**Figure 1:**
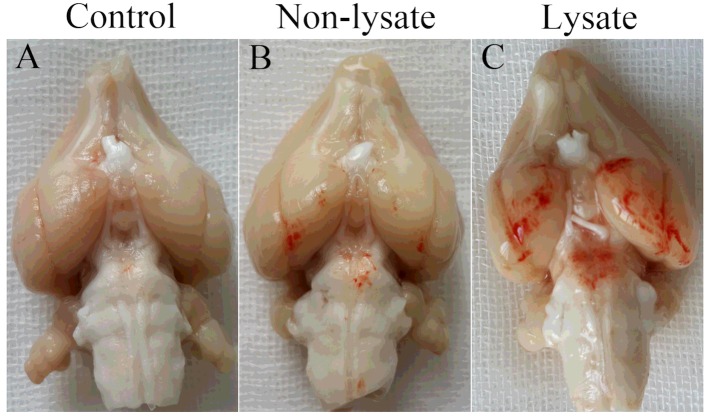
Inferior view of rabbit brain at 72 h after being induced by subarachnoid erythrocytes or their lysate (**A**) no blood stains; (**B**) not obvious blood stains at the base of temporal lobe and in basal cistern; (**C**) obvious blood stains at the base of temporal lobe and in basal cistern.

Hours after anaesthesia recovery, rabbits in the lysate group appeared to be more drooping and lumbering than that in the control group and the non-lysate group. No rabbit died before perfusion-fixation. There were obvious blood stains at the base of temporal lobe and in basal cistern in the lysate group, but not obvious blood stains in the non-lysate group or no blood stains in control group at 72 h after being induced by subarachnoid erythrocytes or their lysate (Figure 1).

### Evaluation of CVS

**Figure 2:**
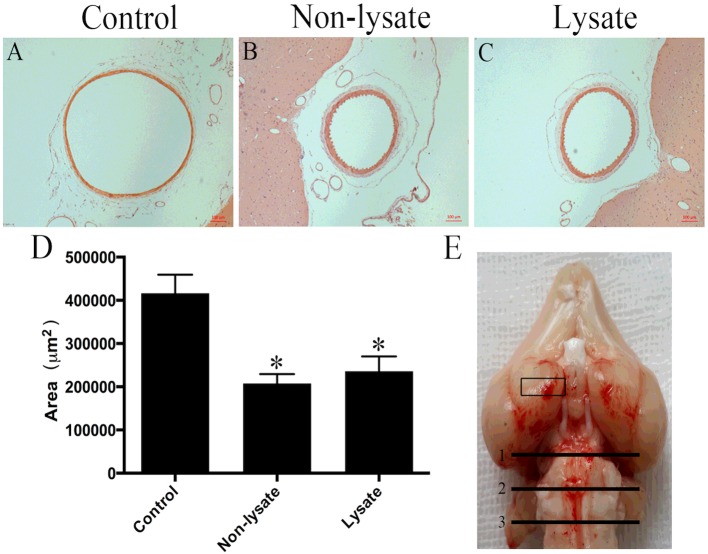
Cerebral vasospasm in rabbits at 72 h after being induced by subarachnoid erythrocytes or their lysate Representative histological cross-sections of the basilar artery in rabbits at 72 h after being induced by subarachnoid erythrocytes or their lysate (**A**–**C**). Mean basilar artery cross-sectional areas (**D**). *n*=6 for each group. Results represent the mean±S.E.M., *P<0.01 compared with the control group. The rectangular part of the rat brain (coronal section) and three segments of the basilar artery (transverse section) were used for histopathological study (**E**).

The representative cross-sections of the basilar artery in each group observed under the light microscope were shown in Figures 2(A)–2(C). The mean basilar artery cross-sectional area in the control group was 416661.4±17508.9 μm^2^. In the non-lysate group and the lysate group, the mean basilar artery cross-sectional areas decreased to 207722.9±8822.6 μm^2^ and 235622.2±12886.7 μm^2^ respectively. This decrease was statistically significant (*P*<0.01). Nevertheless, there was no significant difference in the mean basilar artery cross-sectional areas between the non-lysate group and the lysate group (Figure 2D).

### Evaluation of brain damage

**Figure 3:**
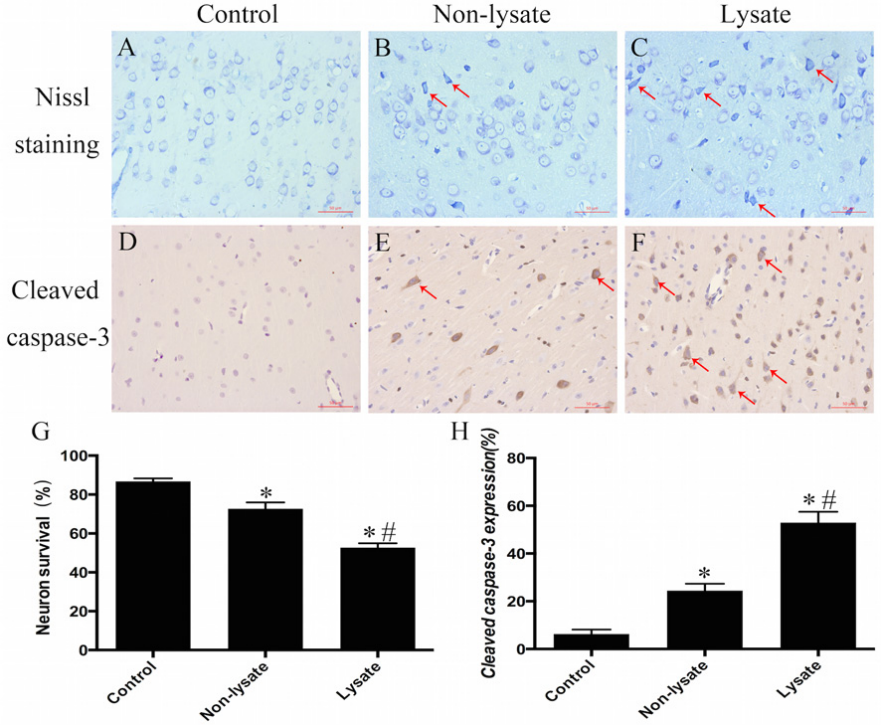
Brain injury in rabbits at 72 h after being induced by subarachnoid erythrocytes or their lysate Nissl staining of cortical tissue sections showed the ratio of neuron survival was lower in the non-lysate group, whereas the lowest in the lysate group (**A**–**C** and **G**). IHC showed the ratio of cleaved caspase-3 positive cells was higher in the non-lysate group, whereas the highest in the lysate group (**D**–**F** and **H**). *n*=6 for each group. Results represent the mean±S.E.M., *P<0.01 compared with the control group, ^#^*P*<0.01 compared with the non-lysate group.

Nissl staining of cortical tissue sections showed the ratio of neuron survival in the non-lysate group was 72.67±1.34%, which was significantly lower than that in the control group (86.80±0.60%; *P*<0.01) and significantly higher than that in the lysate group (52.68±0.94%; *P*<0.01) (Figures 3A–3C and 3G). IHC showed the ratio of cleaved caspase-3 expression in cytoplasm of neural cells in the non-lysate group was 24.50±1.17%, which was significantly higher than that in the control group (6.31±0.77%; *P*<0.01) and significantly lower than that in the lysate group (52.95±1.86%; *P*<0.01) (Figure 3D–3F and 3H).

### Expressions of Prx2, IL-6 and TNF-α

**Figure 4:**
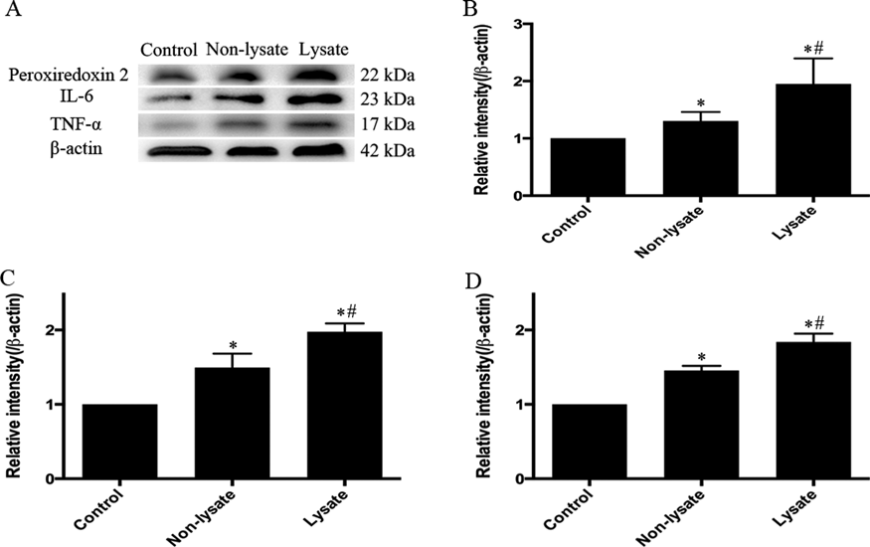
Up-regulation of the expressions of Prx2, IL-6 and TNF-α in the temporal cortex at 72 h after being induced by subarachnoid erythrocytes or their lysate Western blotting analysis showed the expressions of Prx2, IL-6 and TNF-α were increased in the non-lysate group and the lysate group. Moreover, the levels of Prx2 (**B**), IL-6 (**C**) and TNF-α (**D**) in the lysate group were higher than those in the non-lysate group. *n*=6 for each group. Results represent the mean±S.E.M., *P<0.01 compared with the control group, ^#^*P*<0.01 compared with the non-lysate group.

The expressions of Prx2, IL-6 and TNF-α in brain cortex were measured at 72 h after experimental SAH. As shown in Figure 4, up-regulation of Prx2 (Figure 4B), IL-6 (Figure 4C) and TNF-α (Figure 4D) in the temporal cortex was induced by subarachnoid erythrocytes and their lysate. And the expressions of Prx2, IL-6 and TNF-α in the lysate group were significantly higher than those in the non-lysate group (*P*<0.01).

### Biochemical analysis

**Figure 5:**
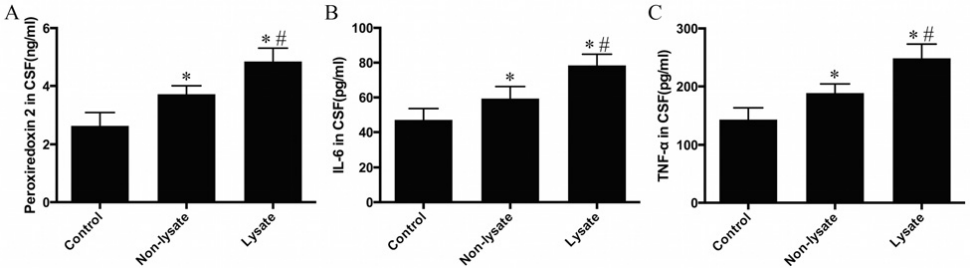
The levels of Prx2, IL-6 and TNF-α in CSF of rabbits at 72 h after being induced by subarachnoid erythrocytes or their lysate ELISA analysis showed the levels of Prx2, IL-6 and TNF-α were simultaneously elevated in the non-lysate group and the lysate group. Moreover, the levels of these proteins in the lysate group were higher than those in the non-lysate group. *n*=6 for each group. Results represent the mean±S.E.M., *P<0.01 compared with the control group, ^#^*P*<0.01 compared with the non-lysate group.

ELISA analysis showed the levels of Prx2, IL-6 and TNF-α in CSF increased at 72 h after experimental SAH (Figures 5A–5C). Among them, the levels of these proteins in the non-lysate group and the lysate group were significantly higher than those in the control group (*P*<0.01), whereas the levels of these proteins in the lysate group were significantly higher than those in the non-lysate group (*P*<0.01).

## DISCUSSION

The mortality rate of patients subjected to SAH was 40–50% in a month [21]. The survivors still bore a high risk of disability [22–25]. Previous research indicated that CVS and EBI were the main factors affecting the prognosis of patients with SAH. It was once thought that the high mortality and disability rates of patients with SAH were mainly caused by CVS. In some clinical trials, prevention of CVS had not improved the prognosis of patients with SAH [26,27]. Besides, some patients with no obvious CVS were found to have severe neurological disorders, suggesting there would be other factors except CVS [28]. Therefore, recent researches were more inclined to be about EBI. The mechanisms of EBI after SAH were studied mainly through the following aspects: primary mechanical damage [29], encephaledema and blood brain barrier damage [30,31],inflammatory injury [32,33], oxidative stress [34,35], toxicity of blood and its lysate [30,36], etc. In these studies, whole blood was usually used as the injury factor, although the lysis of erythrocytes was seldom concerned about.

In clinical practices, it was often found that some complications (such as CVS, cognitive dysfunction, hypothalamic dysfunction, etc.) were still present in the patients with severe SAH after operation of aneurysms [25]. Unfortunately, there were still no effective ways to prevent these complications, as a result of not yet understood mechanisms of these complications. Fortunately, previous studies showed erythrocyte lysate (such as oxygenated haemoglobin, ferri ion, calcium ion, etc.) was of cytotoxicity, which was closely related to brain injury [30]. Based on these results, erythrocyte lysate was supposed to be one of the causes of these complications. Therefore, in order to further study the role of erythrocyte lysate in the subarachnoid space on brain injury, we established the animal model by injecting autologous erythrocytes or its lysate into cistern magna of rabbits.

In this experiment, the animal model was stable and had a high survival rate (no death in this study). Moreover, it was available to detect biochemical indicators in CSF withdrawn from cistern magna of rabbits. The results showed spasm of basilar artery and brain injury (reduction in normal neuron and increase in neural cells with cleaved caspase-3 positive expression) at the bottom of temporal lobe were both observed in the non-lysate group and the lysate group at 72 h after experimental SAH. Moreover, there was not significantly different in spasm of basilar artery between the non-lysate group and the lysate group. Nevertheless, brain injury in the lysate group was significantly more severe than that in the non-lysate group. From the above results, it could be inferred that spasm of basilar artery was not the major factor causing brain injury in this experiment. In addition, up-regulation of the expressions of Prx2, IL-6 and TNF-α in brain cortex and in CSF was detected at 72 h after experimental SAH. The degree of inflammatory response positively correlated with Prx2 expression in the lysate group was more significant than that in the non-lysate group. In short, rabbits in the lysate group subjected to a more destructive inflammatory response and a higher risk of brain injury, and the former was likely to be one of the main causes to the latter.

It had been confirmed that erythrocyte lysate could cause CVS [6,14,37–39] and the mechanisms included oxidative stress [40], inflammatory response [11,41–43], etc. The results of the lysate group were consistent with that in the literature. It was confusing that CVS was also observed in the non-lysate group, which seemed to be inconsistent with the previous report [44]. From the literature we knew, the rate of spontaneous haemolysis was initially slow (approximately 1%/day), and then became more rapid after a 4-day incubation (at 37°C in an artificial CSF) [14]. It might be possible that aging and lysis of erythrocytes accelerated and arose in advance in the subarachnoid space due to some certain conditions change, which finally led to spasm of basilar artery.

As expected, it was confirmed that the level of subarachnoid Prx2 was elevated at 72 h after being induced by subarachnoid erythrocytes and their lysate in this experiment. And what was the role of Prx2? For one thing, as one of the most abundant proteins in erythrocytes after haemoglobin, Prx2 may cause brain injury, like haemoglobin in the previous study [2], inducing release of high-mobility group box 1 (HMGB1) and leading to EBI through the Toll-like receptor (TLR) mediated inflammatory response. For another, Shichita et al. [45] found Prxs released from damaged neural cells after stroke, lost their neuroprotective function and initiated activation of nuclear factor-κB (NF-κB) in these immune cells and then led to production of proinflammatory cytokines, which finally triggered a destructive inflammatory response. Prxs played a key role in proinflammatory response. As Garcia-Bonilla and Iadecola [12] suggested, Prxs set the brain on fire after stroke. In this experiment, the levels of Prx2 and inflammatory cytokines (IL-6, TNF-α) were simultaneously elevated after experimental SAH and peaked in the lysate group with the most severe brain injury. Therefore, the results suggested that the inflammatory response positively correlated with Prx2 might be involved in the mechanism of brain injury.

However, there were still some aspects not yet understood. Firstly, were the roles of Prx2 from these two different sources consistent? Secondly, what kind of source contributed more to elevation of Prx2 level? Thirdly, was there a vicious circle that Prx2, as a crucial link in the chain of events after SAH, initiated a destructive inflammatory response leading to brain injury and intracellular Prx2 in damaged neuron was then released, which eventually caused further damage? Fourthly, after SAH, which pathway did Prx2 play a role through? The TLR2/4 - NF-κB pathway? What kind of inhibitor could be chosen to block the pathway? Fifthly, in addition to the destructive inflammatory response, cortical neurons might be subjected to cytotoxicity of erythrocyte lysate and oxidative stress, etc.

There were also some clinical implications. First of all, these results in this experiment suggested that some delayed neurological disorders might be caused by subarachnoid erythrocyte lysate, which should deserve more attention in clinic practices despite of treatment of ruptured aneurysms. The next, some patients of severe SAH with a low Glasgow Coma Scale score and a high Fischer scale score was badly sick, worsened rapidly, and even died in a short time. In addition to some known causes (such as intracranial hypertension, etc.), the massive lysis of subarachnoid erythrocytes may be possible to cause the consequence due to fragile brittleness of erythrocytes themselves or free into CSF. Besides, extracellular Prx2 may act as a crucial link in the inflammatory response after SAH. The level of Prx2 in CSF was positively correlated with the degree of brain injury and would be used as an indicator of prognostic analysis. By inhibiting or blocking the role of extracellular Prx2 with some specific antibodies, it would be possible to reduce the inflammatory response and play a neuroprotective role, leading to improve the prognosis. And all above need further study.

## Abbreviations

CSF: cerebrospinal fluid
CVS: cerebral vasospasm
DAB: 3,3′-diaminobenzidine
DCI: delayed cerebral ischemia
EBI: early brain injury
H_2_O_2_: hydrogen peroxide
HMGB1: high-mobility group box 1
IHC: immunohistochemistry
NF-κB: nuclear factor-κB
Prx2: peroxiredoxin 2
Prxs: peroxiredoxins
SAH: subarachnoid haemorrhage
TLR: Toll-like receptor
